# A trial deep learning-based model for four-class histologic classification of colonic tumor from narrow band imaging

**DOI:** 10.1038/s41598-023-34750-3

**Published:** 2023-05-09

**Authors:** Takeshi Shimizu, Yoshihiro Sasaki, Kei Ito, Masashi Matsuzaka, Hirotake Sakuraba, Shinsaku Fukuda

**Affiliations:** 1 Department of Gastroenterology, Sendai City Medical Center Sendai Open Hospital, 5-22-1 Tsurugaya, Miyagino-ku, Sendai, 983-0824 Japan; 2 Department of Medical Informatics, Hirosaki University Hospital, 53 Hon-cho, Hirosaki, 036-8563 Japan; 3 Department of Gastroenterology and Hematology, Hirosaki University Graduate School of Medicine, 5 Zaifu-cho, Hirosaki, 036-8562 Japan; 4 Department of Community Medical Support, Hirosaki University Graduate School of Medicine, 5 Zaifu-cho, Hirosaki, 036-8562 Japan

**Keywords:** Computational biology and bioinformatics, Computational models

## Abstract

Narrow band imaging (NBI) has been extensively utilized as a diagnostic tool for colorectal neoplastic lesions. This study aimed to develop a trial deep learning (DL) based four-class classification model for low-grade dysplasia (LGD); high-grade dysplasia or mucosal carcinoma (HGD); superficially invasive submucosal carcinoma (SMs) and deeply invasive submucosal carcinomas (SMd) and evaluate its potential as a diagnostic tool. We collected a total of 1,390 NBI images as the dataset, including 53 LGD, 120 HGD, 20 SMs and 17 SMd. A total of 598,801 patches were trimmed from the lesion and background. A patch-based classification model was built by employing a residual convolutional neural network (CNN) and validated by three-fold cross-validation. The patch-based validation accuracy was 0.876, 0.957, 0.907 and 0.929 in LGD, HGD, SMs and SMd, respectively. The image-level classification algorithm was derived from the patch-based mapping across the entire image domain, attaining accuracies of 0.983, 0.990, 0.964, and 0.992 in LGD, HGD, SMs, and SMd, respectively. Our CNN-based model demonstrated high performance for categorizing the histological grade of dysplasia as well as the depth of invasion in routine colonoscopy, suggesting a potential diagnostic tool with minimal human inputs.

## Introduction

Although it is relatively simple for human observers to recognize and describe the visual elements in empirical terms, it has been remarkably difficult to accurately define and analyze them with a computer. The electronic endoscope has allowed us to quantify any element making up a digitized endoscopic image through mathematical processes. Several studies have evaluated the effectiveness of feature extraction for computer-aided diagnosis (CAD) to classify the endoscopic severity of ulcerative colitis^1,2^ and to assess the risk of developing gastric cancer among Helicobacter pylori-positive patients^3^. However, the diagnostic accuracy of feature engineering was limited due to the challenges in extracting features for image analysis in gastrointestinal diseases.

With the proliferation of CNN, the task of classifying objects in natural images can be solved simply by presenting examples of images and the names of the objects to a neural network that acquired all its knowledge from the training data^4^. This groundbreaking technology has freed engineers from feature engineering and endoscopists from knowledge-based image interpretation. The CNN-based supervised learning has been applied in the automated localization of gastric cancer in routine gastroscopies^5^ and the automated detection of colon polyps^6^. Several studies have reported the utilization of a CNN-based model for distinguishing adenomatous from non-adenomatous polyps^7^, adenomatous from hyperplastic diminutive colorectal polyps ^8^, and neoplastic polyps from non-neoplastic polyps ^9^. The fine-tuning of a pre-trained CNN for gastric precancerous disease classification^10^ or the efficient channel attention deep dense CNN for the classification of esophageal disease^11^ has also been reported.

However, to the best of our knowledge, a multi-class model for evaluating the grade of histologic dysplasia along with the depth of invasion has not yet been developed. The NBI international colorectal endoscopic (NICE) classification applicable to with or without magnification was proposed for diagnosing submucosal invasive colon cancer^12^. However, criteria for classification described in empirical terms^12^ may inevitably suffer from a variety of biases in evaluation leading to different accuracy varying with endoscopists and disturb comparison of accuracy among different endoscopist communities. This study aimed to develop a trial CNN-based supervised learning model for evaluating histologic atypism or invading depth from NBI images of detected colonic neoplastic lesions and evaluate the potential as a diagnostic tool.

## Methods

### Preparation of endoscopic images

NBI images of neoplastic lesions from patients who underwent endoscopic or surgical resection at Sendai City Medical Center Sendai Open Hospital from April 2017 to December 2019 were used for this single center retrospective study. Characteristics of collected NBI images are summarized in Table 1. A total of 1390 NBI images were sampled from a total of 210 lesions with definite histologic diagnosis^13^: 53 low-grade dysplasia (LGD); 120 high grade dysplasia or mucosal carcinoma (HGD); 20 superficially invasive (the depth of the invasive front < 1000 µm) submucosal carcinoma (SMs) and 17 deeply invasive (the depth of the invasive front > 1000 µm) submucosal carcinomas (SMd). Pathological diagnosis was conducted by pathologists unaware of the study design in a blinded manner. The diagnosis of a mucosal lesion, LGD or HGD was assigned to the most severe grade regardless of the size of the component. Sampled picture number per lesion was 5.5 to 7 samples with an averaged image capturing conditions: no magnification 41.0%; low magnification 37.9%; high magnification 21.1%. The images of a solitary lesion at varying magnifications were carefully chosen to minimize potential bias in the selection process. The video endoscopes CF-HQ290ZI, PCF-H290ZI, PCF-H290TI and video endoscopy system EVIS LUCERA ELITE CV-290/CLV-290SL (Olympus Medical Systems, Co., Ltd., Tokyo, Japan) were used.

**Table 1:** Collected NBI images for dataset. NBI, narrow band imaging; HGD, high grade dysplasia; LGD, low grade dysplasia; SMs, superficially invasive submucosal carcinoma; SMd, deeply invasive submucosal carcinoma.

Histology	Number of lesions	Number of still pictures	Averaged picture number per lesion	Magnification (picture counts/ %)		
				None	Low	High
LGD	53	294	5.5	126/42.9	120/40.8	48/16.3
HGD	120	840	7.0	345/41.1	305/36.3	190/22.6
SMs	20	138	6.9	59/42.8	46/33.3	33/23.9
SMd	17	118	6.9	40/33.9	56/47.5	22/18.6
total	210	1390	6.6	570/41.0	527/37.9	293/21.1

### Preparation of dataset

**Figure 1:**
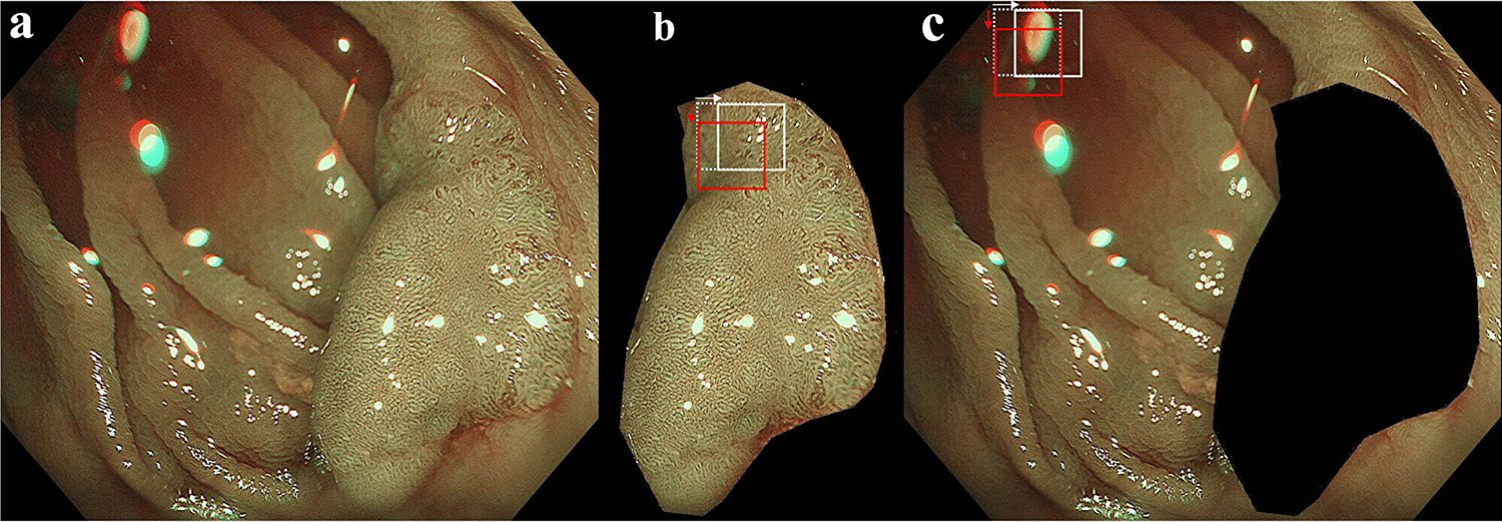
Preparation of dataset. Original NBI image (**a**) were manually partitioned into the lesion (**b**) and background (**c**). The patch images (128 × 128 pixels) were trimmed from the lesion and background starting from the left upper corner (white dotted patch), rightwards (white solid patch), then downwards (red solid patch) at every 32-pixel-strides (white and red arrows) over the entire region of interest.

NBI images (Fig. 1a) were manually partitioned into the lesion (Fig. 1b) and background (Fig. 1c) from which the patch images (128 × 128 pixels) were cropped starting from the left upper corner (white dotted patch), rightwards (white solid patch), then downwards (red solid patch) at every 32-pixel-strides (white and red arrows) over the entire effective region of interest. The patches including blackouts with more than 10% of the effective region were automatically excluded from analysis. Blackouts were defined as regions with the intensity of red component lower than 50. Similarly, the patches with halations exceeding 5% of the effective region were also excluded. Halations were defined as regions with the intensity of green component higher than 250. In this study, the patches were further classified into in-focus patches and out-of-focus ones according to the amount of spatial high frequency area extracted by high pass filter with a cut-off of 6.25% Nyquist frequency. The in-focus patches were classified into (0) background (BG), (1) LGD, (2) HGD, (3) SMs and (4) SMd, and the out-of-focus ones into (5) background (BG-oof) and 6) lesion (L-oof). A total of 598,801 patches were classified into 7 categories (Table 2). The study did not have any inclusion or exclusion criteria for pictorial quality of the patches by endoscopists. As stated, the patches with excessive blackout or halation were automatically excluded before entry. The study aimed to establish an effective histologic classifier that can be used in any common shooting conditions of NBI.

**Table 2:** Quantity of clipped patches within each category. BG, background; HGD, high grade dysplasia; LGD, low grade dysplasia; SMs, superficially invasive submucosal carcinoma; SMd, deeply invasive submucosal carcinoma; BG-oof, out-of-focus background; L-oof, out-of-focus lesion.

Category	The number of patches
BG	91,571
LGD	52,184
HGD	158,187
SMs	28,049
SMd	20,882
BG-oof	167,081
L-oof	80,847

### Evaluation method

We employed cross-validation to obtain more accurate results with less bias in the machine learning studies^14^. In this study, the dataset is randomly partitioned into three equal sized folds, one fold of which is for validation and the other folds are for training. The proportion of labels was equal in each fold. The training and validation processes were repeated three times using different folds each time. The three validation results could then be averaged to produce a single estimation.

### Architecture of the CNN

ResNet50 (a CNN) proposed by He et al.^15^ and Pytorch were utilized. ResNet50 without pretraining was imported from Pytorch library (torchvision.models). The original patches with 128 × 128 pixels were converted into images with 224 × 224 pixels. We tuned hyper parameters, which were set by a human, as follows: optimizer, Adam; loss function, cross entropy loss; number of training epochs, 50; batch size, 256; learning rate, 0.00005 via trial and error; and number of the outer layers, 7 classes.

### Image-level classification

**Figure 2:**
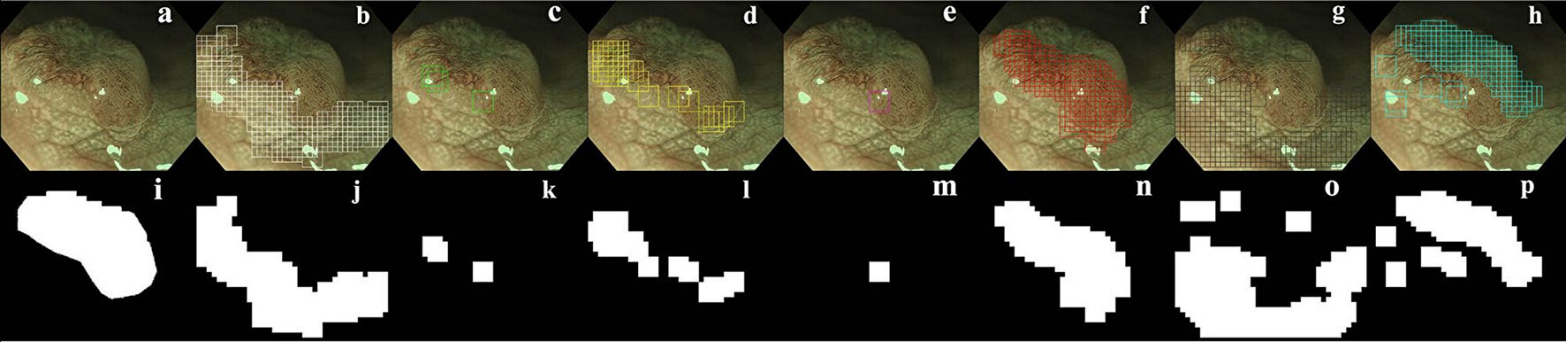
The patch-level histological mapping predicted by the trained model, alongside the annotation mask. An example picture of SMd and the annotation mask without blackout or halation can be seen in (**a**) and (**i**), respectively. White (**b**), green (**c**), yellow (**d**), magenta (**e**), red (**f**), dark gray (**g**), and cyan (**h**) open squares indicate the patches classified as BG, LGD, HGD, SMs, SMd, BG-oof, and L-oof, respectively, with their corresponding union masks visible in (**j–p**), respectively. BG, background; HGD, high grade dysplasia; LGD, low grade dysplasia; SMs, superficially invasive submucosal carcinoma; SMd, deeply invasive submucosal carcinoma; BG-oof, out-of-focus background; L-oof, out-of-focus lesion.

An exemplification of SMd and the annotation mask without blackout or halation (denoted by X) are depicted in Fig. 2a,i, respectively. The patches classified into BG, LGD, HGD, SMs, SMd, BG-oof and L-oof, by the trained CNN, are illustrated by white (Fig. 2b), green (Fig. 2c), yellow (Fig. 2d), magenta (Fig. 2e), red (Fig. 2f), dark gray (Fig. 2g) and cyan (Fig. 2h) open squares, respectively, and the corresponding union masks in Fig. 2j–p, respectively. Classification algorithms must be developed by utilizing in-focus patches, without sacrificing pictorial information. Here, the union masks of the patches classified into labels BG, LGD, HGD, SMs and SMd are designated by M0, M1, M2, M3 and M4, respectively. Intersection over union between X and Mi (IoUi) are given by X ∩ Mi/ X ∪ Mi (i = 0, 1, 2, 3, 4). The lesion was classified into the argmax among IoUi (i = 0, 1, 2, 3, 4), with the IoUi values of 0.12, 0.05, 0.21, 0.04 and 0.57, respectively, leading to label 4 or histologic classification SMd.

### Ethics approval and consent to participate

This study was approved by the Committee of Medical Ethics of Hirosaki University Graduate School of Medicine (Aomori, Japan; reference no. 2019–1099) and Sendai City Medical Center (Sendai, Japan: reference no. 2019–0029). Informed consent was obtained in the form of opt-out on our website (https://www.https://www.med.hirosaki-u.ac.jp/hospital/outline/resarch.html), with the approval of the Committee of Medical Ethics of Hirosaki University Graduate School of Medicine. This study was designed and carried out in accordance with the Declaration of Helsinki.

## Results

### Accuracy of patch-level and image-level classification

An averaged validation accuracy in the patches with label 0, 1, 2, 3, 4, 5 and 6 was 0.938, 0.876,0.957, 0.907, 0.929, 0.966 and 0.904, respectively (Table3). Table 4 displays the confusion matrix diagram depicting the outcomes of the image-level classification from the patch-based mapping across the entire image area using the trained CNN. The ground truth histology was located on the vertical axis, and the predicted histology was situated on the horizontal axis. Out of 1,390 pictures, 1,371 pictures were correctly classified into the correct histology with a total accuracy of 0.986. The precision and F1-scores were determined to be 0.973, 0.992, 1, 0.967 and 0.978, 0.991, 0.982, 0.980 for LGD, HGD, SMs, and SMd, respectively.

**Table 3:** Patch-level three-fold validation accuracy. BG, background; HGD, high grade dysplasia; LGD, low grade dysplasia; SMs, superficially invasive submucosal carcinoma; SMd, deeply invasive submucosal carcinoma; BG-oof, out-of-focus background; L-oof, out-of-focus lesion.

Category	Fold 1			Fold 2			Fold 3			Averaged accuracy
	Total patches	Correct patches	Accuracy	Total patches	Correct patches	Accuracy	Total patches	Correct patches	Accuracy	
BG	30,524	28,634	0.938	30,524	28,625	0.938	30,523	28,637	0.938	0.938
LGD	17,395	15,011	0.863	17,395	15,044	0.865	17,394	15,644	0.899	0.876
HGD	52,729	50,383	0.956	52,729	50,327	0.954	52,729	50,703	0.962	0.957
SMs	9350	8616	0.921	9350	8657	0.926	9349	8163	0.873	0.907
SMd	6961	6115	0.878	6961	6632	0.953	6960	6659	0.957	0.929
BG-oof	55,694	54,108	0.972	55,694	53,855	0.967	55,693	53,386	0.959	0.966
L-oof	26,949	24,664	0.915	26,949	23,758	0.882	26,949	24,695	0.916	0.904

**Table 4:** Confusion matrix and image-level accuracy. BG, background; HGD, high grade dysplasia; LGD, low grade dysplasia; SMs, superficially invasive submucosal carcinoma; SMd, deeply invasive submucosal carcinoma.

Ground truth	Predicted histology				total	Recall
	LGD	HGD	SMs	SMd		
LGD	289	4	0	1	294	0.983
HGD	6	832	0	2	840	0.990
SMs	2	2	133	1	138	0.964
SMd	0	1	0	117	118	0.992

### Examples of the patch-based mapping and image-level classification

**Figure 3:**
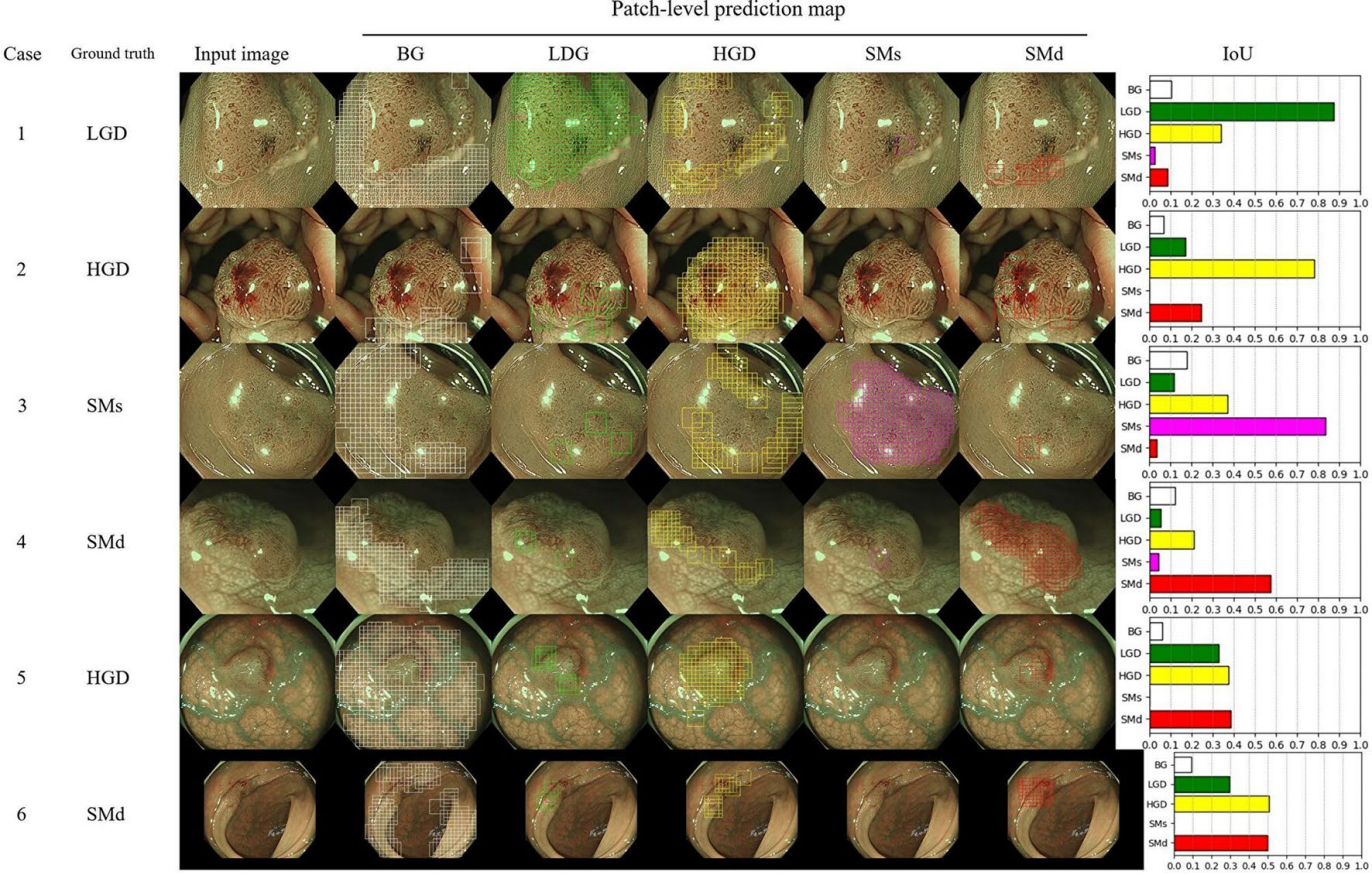
Input images with patch-level prediction map and IoU. BG, background ; HGD, high grade dysplasia ; LGD, low grade dysplasia ; SMs, superficially invasive submucosal carcinoma ; SMd, deeply invasive submucosal carcinoma; IoU, intersection over union.

Figure 3 illustrates the examples of input images, patch-level prediction map and bar graph of IoU. In cases 1, 2, 3 and 4, the ground truth histology was consistent with the predicted histology with the maximum intersection over union. In cases 5 and 6, HGD and SMd were misclassified as SMd and HGD, respectively. In these cases, misclassification of the surrounding background into the true lesion resulted in a lower intersection over union of the true lesion compared to the misclassified ones. A type of misclassification, stemming from an underestimation of the actual lesion compared to misclassified lesions across four SMs, has likely caused a decrease in accuracy relative to other lesions.

## Discussion

In this study, we developed a trial CNN-based multi-class histology classifier model for detected colorectal neoplastic lesion in routine colonoscopy still images with NBI mode in common shooting condition. The NBI offers a significant advantage for CNN-based image classification thanks to its ability to provide high contrast or detailed pictorial information without requiring any pre-acquisition preparation. The diagnosis process includes patch-level histology mapping over the entire in-focus region of NBI image, trained on ResNet50 and the calculation of argmax among intersections over union between annotation mask and patch-level union masks for image-level histology. This model achieved an image-level accuracy of 0.986, suggesting its potential as a diagnostic tool.

The advancement of machine learning using CNN has enabled physicians to apply CAD of medical images in their specialized field. The American Society for Gastrointestinal Endoscopy AI Task Force^16^ stated that CAD plays a crucial role in screening and surveillance colonoscopy for colorectal cancer prevention. Similarly, a European Society of Gastrointestinal Endoscopy mentioned to the capability of AI for accurately predicting the histology of polyps from endoscopic images and improving the cost-efficiency and safety of colonoscopic colorectal cancer screening and surveillance^17^. The supervised learning of a CNN has enabled the development of a model for automated detection of colon polyps^6^, as well as binary classification models for distinguishing adenomatous from non-adenomatous polyps (with a ten-fold validation accuracy of 0.751)^7^, adenomatous from hyperplastic diminutive colorectal polyps (with an accuracy of 94%)^8^, and neoplastic polyps from non-neoplastic polyps (with a high confidence rate of 0.85)^9^.

However, a CNN model for multiclass differentiation among low-grade dysplasia, high-grade dysplasia, and carcinoma with superficial or deep submucosal invasion has not been developed. Although invading depth is determinant for therapeutic intervention (endoscopic resection or surgery), it has been evaluated so far by endoscopists with the use of knowledge-based criteria^12,18^ which inevitably suffers from a variety of biases in evaluation. One study has reported a CNN-based binary class prediction model for deeply submucosal invasive carcinoma with an overall accuracy of 85.5%, which is comparable to that of expert endoscopists^19^. Although it must be done with caution, comparison of model accuracy between studies with different designs revealed a prediction accuracy of 0.991 for carcinoma with deeply submucosal invasion in this study. To accurately compare the accuracy of ML models regardless of algorithm and class numbers, the promotion of a benchmark data set library with annotation masks^20^ is essential.

A patch-based CNN has been utilized for automated detection of a target area within a whole slide image in digital pathology^21,22^. This method has been recently applied for automated severity mapping along the entire colorectum in patients with ulcerative colitis from capsule endoscopy video files^23^, as it is considered to have an advantage when the object for classification is composed of topographically varying elements, such as severity or atypism. When reconstructing histologic maps in resected specimens, one often encounters topographic heterogeneity in the grade of dysplasia as well as invading depth. In this study, a single histology or label was assigned to a single lesion image, thus resulting in similar labels across the entire lesion area, which may have impacted the outcomes. None the less, a multi-class classification model developed from a trained patch-level classifier has achieved a high image-level accuracy of over 0.96, which may provide a potential diagnostic tool with minimal human input in routine colonoscopy.

The limitations of the study were: its single-center, retrospective nature, limited dataset size, and lack of external validation; moreover, other classification models including VGGNets, DenseNets and ViT were not explored; the applicability of the model to diagnosis in the subsequent endoscopy system is uncertain.

## Data Availability

The data generated or analyzed during this study are included in this published article. Some datasets generated and/or analyzed during the current study are not publicly available due to privacy but are available from the corresponding author on reasonable request.
